# Comparative Diagnostic Accuracy of Ganglion Cell-Inner Plexiform and Retinal Nerve Fiber Layer Thickness Measures by Cirrus and Spectralis Optical Coherence Tomography in Relapsing-Remitting Multiple Sclerosis

**DOI:** 10.1155/2014/128517

**Published:** 2014-09-18

**Authors:** Julio J. González-López, Gema Rebolleda, Marina Leal, Noelia Oblanca, Francisco J. Muñoz-Negrete, Lucienne Costa-Frossard, José C. Álvarez-Cermeño

**Affiliations:** ^1^Department of Ophthalmology, Ramón y Cajal University Hospital, Carretera de Colmenar Km 9, 1, 28034 Madrid, Spain; ^2^Department of Surgery, Alcalá de Henares University, Madrid, Spain; ^3^Medical Retina Department, Moorfields Eye Hospital, London, UK; ^4^Department of Neurology, Ramón y Cajal University Hospital, Madrid, Spain

## Abstract

*Objective*. To estimate sensitivity and specificity of several optical coherence tomography (OCT) measurements for detecting retinal thickness changes in patients with relapsing-remitting multiple sclerosis (RRMS), such as macular ganglion cell-inner plexiform layer (GCIPL) thickness measured with Cirrus (OCT) and peripapillary retinal nerve fiber layer (pRNFL) thickness measured with Cirrus and Spectralis OCT.* Methods*. Seventy patients (140 eyes) with RRMS and seventy matched healthy subjects underwent pRNFL and GCIPL thickness analysis using Cirrus OCT and pRNFL using Spectralis OCT. A prospective, cross-sectional evaluation of sensitivities and specificities was performed using latent class analysis due to the absence of a gold standard.* Results*. GCIPL measures had higher sensitivity and specificity than temporal pRNFL measures obtained with both OCT devices. Average GCIPL thickness was significantly more sensitive than temporal pRNFL by Cirrus (96.34% versus 58.41%) and minimum GCIPL thickness was significantly more sensitive than temporal pRNFL by Spectralis (96.41% versus 69.69%). Generalised estimating equation analysis revealed that age (*P* = 0.030), optic neuritis antecedent (*P* = 0.001), and disease duration (*P* = 0.002) were significantly associated with abnormal results in average GCIPL thickness.* Conclusion*. Average and minimum GCIPL measurements had significantly better sensitivity to detect retinal thickness changes in RRMS than temporal pRNFL thickness measured by Cirrus and Spectralis OCT, respectively.

## 1. Introduction

Relapsing-remitting multiple sclerosis (RRMS) is a chronic, immune-mediated demyelinating disease of the central nervous system that frequently involves the visual pathways, usually in the form of optic neuritis (ON) [1]. Postmortem analysis revealed optic nerve lesions in 94–99% of RRMS patients, even in the absence of a clinical history of ON [2].

Optical coherence tomography (OCT) is a noninvasive and reproducible tool for evaluating the retinal and optic disc anatomy of patients with this clinical disorder. It uses low-coherence interferometry to obtain detailed images of the retinal architecture. Modern high-speed spectral-domain (SD) OCT devices can obtain high resolution images of the retina. Computerised algorithms can be used on these images in order to automatically identify and obtain thickness measurements of discrete retinal layers, including the retinal nerve fiber layer (RNFL) and the macular ganglion cell-inner plexiform layers (GCIPL) [3].

In patients with RRMS, the main focus has been the evaluation of the peripapillary retinal nerve fiber layer (pRNFL). The RNFL contains the unmyelinated axons originating from the ganglion cell neurons. In a previous report, we found that Spectralis showed a significantly higher thinning for temporal quadrant than Cirrus in eyes of RRMS patients, suggesting that N-site axonal analysis could define axonal damage in relapsing-remitting multiple sclerosis patients earlier than conventional pRNFL analysis [4].

Optic nerve demyelination, due to clinical or subclinical ON, can result in retrograde degeneration of the optic nerve axons, leading to RNFL and GCIPL thinning [5, 6]. In fact, several studies have reported statistically significant thinning of the pRNFL and GCIPL in patients with RRMS with and without optic neuritis compared to healthy control subjects [7–10].

Moreover, macular GCIPL thickness has been found to have better structure-function correlations than pRNFL thickness with both visual function and disability in RRMS patients [11]. At least in part, this observation may be due to the superior reproducibility of GCIPL over pRNFL thickness measurements [11].

Thus, we hypothesize that GCIPL measurements can be more sensitive to and specific of retinal involvement in patients with RRMS than those with pRNFL measurements.

The main purpose of this study was to estimate the sensitivity and specificity of macular GCIPL thickness measured with the Cirrus OCT ganglion cell analysis (GCA) algorithm (Carl Zeiss Meditec AG, Jena, Germany) and pRNFL thickness analysis with Cirrus and Spectralis (Heidelberg Engineering GmbH, Heidelberg, Germany) OCTs in detecting retinal thickness changes in eyes from patients with a clinical diagnosis of RRMS versus age-matched normal subjects using latent class analysis.

Ancillary objectives were to quantify color-code abnormalities in GCIPL and pRNFL measures by Cirrus and Spectralis OCTs in eyes from patients with RRMS and healthy control subjects, to quantify the correlations of GCIPL and pRNFL measurements with the visual function and disability in MS patients, and to study the effect of optic neuritis antecedent on the obtained measurements.

## 2. Methods

### 2.1. Subjects

An observational, prospectively recruited, cross-sectional study was performed. The study was approved by the Research Ethics Committee in the Ramón y Cajal University Hospital. All research complied with the tenets of the declaration of Helsinki, and all subjects participating in the study gave their written informed consent. Confidentiality of participating subjects was protected throughout the study.

We included 70 patients with a diagnosis of RRMS and 70 healthy control subjects. Patients were enrolled consecutively from the Neuroophthalmology Department from January 2012 to September 2012. Healthy controls without a history of neurological and ophthalmological disease were recruited among the hospital staff.

Diagnosis of RRMS was based on McDonald criteria by the treating neurologist [12]. None of the included patients had a diagnosis of secondary progressive multiple sclerosis.

All participants underwent a complete neuroophthalmic evaluation that included pupillary, anterior segment, and funduscopic examinations; assessment of logMAR best corrected visual acuity (BCVA), and they were scanned after pupillary dilation with Cirrus (Carl Zeiss Meditec AG, Jena, Germany) and Spectralis (Heidelberg Engineering GmbH, Heidelberg, Germany) OCTs on the same day in random order. Both eyes of each subject were included. Exclusion criteria were intraocular pressure of 21 mm Hg or higher, an optic disc suspicious for glaucoma under dilated funduscopy, a refractive error greater than 5.0 diopters (D) of spherical equivalent or 3.0 D of astigmatism in either eye, media opacity, a recent history of optic neuritis 6 months prior to the day of imaging, systemic conditions that could affect the visual system, a history of ocular trauma, or concomitant ocular diseases, including glaucoma.

Related medical records were carefully reviewed, including the duration of the disease, the Expanded Disability Status Scale (EDSS) scored by a neurologist (LC), and the presence of prior episodes of optic neuritis (ON) as reported by the treating neurologist and the patient.

The visual field (VF) was tested only in the eyes of patients with RRMS, using a Humphrey Field Analyzer (Carl Zeiss Meditec AG, Jena, Germany) and the SITA Standard protocol (program 24-2). VF test was considered reliable when fixation losses were less than 20% and false-positive and false-negative errors were less than 15%.

### 2.2. Optical Coherence Tomography Measurements

A single, well-trained optometrist (NO) performed all OCT examinations in random order to prevent any fatigue bias. All poor-quality scans were rejected, defined as those with signal strength of ≤6 by Cirrus. For Spectralis OCT only those images with a signal-to-noise score higher than 25 dB were analyzed. Scans with misalignment, segmentation failure, decentration of the measurement circle, and poor illumination or those out of focus were excluded from the analysis. Thus, manual correction of plotting errors of automated segmentation was not performed in this study.

Methodology for pRNFL imaging in Cirrus and Spectralis has been reported previously [3]. Briefly, cross-sectional imaging of the peripapillary area was performed using Cirrus OCT. pRNFL thickness was determined using the optic disc cube protocol (software version 5.1.1.6) that generates a cube of data through a 6 mm square grid. A 3.46 mm in diameter calculation circle was automatically positioned around the optic disc. Cirrus OCT provides average pRNFL thickness and maps with 4 quadrants (superior, inferior, nasal, and temporal) and 12 clock hours, including classification from an internal normative database.

Spectralis OCT (software version 5.2.0.3) simultaneously captures infrared fundus and SD-OCT images at 40,000 A-scans per second. A real-time eye-tracking system measures eye movements and provides feedback to the scanning mechanism to stabilize the retinal position of the B-scan. The instrument uses 1024 A-scan points from a 3.45 mm circle centered on the optic disc. The examiner is required to manually place the scan around the optic disc. Peripapillary RNFL measurements were obtained using the N-site axonal protocol, which differs from the standard pRNFL scan because it starts and terminates in the nasal side of optic nerve. Scans were obtained using the high resolution (HR) mode and using automatic real-time (ART) for averaging 9 B-scan frames in order to improve image quality. The pRNFL Spectralis protocol generates a map showing the average thickness, maps with 4 quadrants (superior, inferior, nasal, and temporal), and maps with 6 sector thicknesses (superonasal, nasal, inferonasal, inferotemporal, temporal, and superotemporal).

The pRNFL thicknesses in the normal range are represented by green backgrounds. Those that are abnormal at the 5% and at the 1% level are represented by yellow and red backgrounds, respectively. The hypernormal (95th to 100th percentiles) pRNFL thicknesses are presented by a white color in Cirrus and by a blue/purple color in Spectralis.

**Figure 1:**
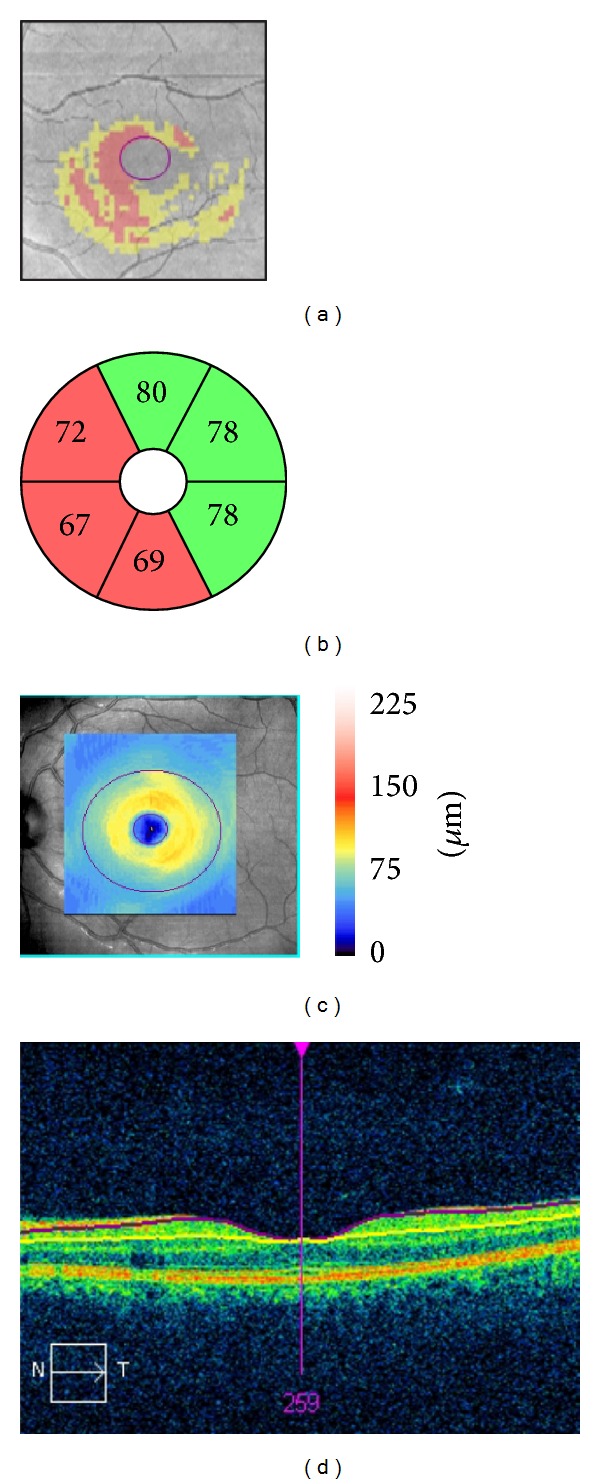
Ganglion cell-inner plexiform layer (GCIPL) analysis of the left eye of a patient with relapsing-remitting multiple sclerosis without optic neuritis antecedent in a 6 × 6 × 2 mm macular cube using a Cirrus optical coherence tomography. (a) Deviation map of the GCIPL thickness (red: below percentile 1; yellow: below percentile 5). (b) Sector distribution. (c) GCIPL thickness map, with overlying ellipses showing the dimensions of the analyzed annulus. The outer ellipse has a vertical diameter of 4 mm and the inner ellipse of 1 mm. (d) Horizontal B-scan centered in the fovea, showing the automated segmentation of the GCIPL.

Cross-sectional imaging of the macular area was performed using Cirrus OCT macular cube (512 × 128). This acquisition protocol generates a cube through a 6 mm square grid of 128 B-scans of 512 A-scans each. A built-in GCIPL analysis algorithm detects and measures the thickness of the macular GCIPL within a 6 × 6 × 2 mm elliptical annulus area centered on the fovea. The annulus has an inner vertical diameter of 1 mm, which was chosen to exclude the portions of the fovea where the layers are very thin and difficult to detect accurately, and an outer vertical diameter of 4 mm, which was chosen according to where the GCL again becomes thin and difficult to detect. The GCA algorithm identifies the outer boundaries of the RNFL and IPL. The difference between the RNFL and the IPL outer boundary segmentations yields the combined thickness of the RGC layer and IPL. Cirrus OCT provides quantitative assessment of the ganglion cell and inner plexiform layers (GCIPL) in 6 circular sectors centered in the fovea (superonasal, superior, inferonasal, inferotemporal, inferior, and superotemporal). It also gives information on the mean and minimum GCIPL thickness for each eye and compares these figures with a normative database (Figure 1). The GCIPL thicknesses in the normal range are represented by green backgrounds. Those that are abnormal at the 5% and at the 1% level are represented by yellow and red backgrounds, respectively. The hypernormal (95th to 100th percentiles) pRNFL thicknesses are presented by a white color.

### 2.3. Statistical Analysis

Data were analyzed using Stata/SE 12.0 for Unix and IBM SPSS Version 20 for Unix. A *P* value of less than 0.05 was considered statistically significant.

Quantitative variables were summarized as mean ± standard deviation. Qualitative variables were summarized as absolute value (percentage). Generalized estimating equation models accounting for sex, age, and within-patient intereye correlations were used to examine correlations and associations between variables.

When evaluating new medical diagnostic tests, data may be obtained from one or more tests, but none of these can be considered a gold standard, that is, a diagnostic test with 100% sensitivity and specificity [13]. Latent class analysis (LCA) is based on the concept that observed results of different imperfect tests for the same disease are influenced by a latent common variable, the true disease status, which cannot be directly measured. In a group of patients with unknown disease status, for whom results from several diagnostic tests are available, LCA will model the probability of each combination of test results on the latent class and will provide an estimate of sensitivity and specificity for each of the diagnostic tests evaluated [14, 15]. LCA has been used extensively for the estimation of sensitivity and specificity of diagnostic tests in the absence of a valid gold standard, mainly in microbiology [16, 17] and psychology [18], but also in ophthalmology [19].

In this study, we implemented the basic latent class model, using the assumption of conditional independence given the latent class. In basic LCA, there are no associations between the observed variables within each category of the latent variable. The latent variable is the true status on the disease, and the hypothesis is that there are two latent classes (presence or absence of retinal thickness changes). As more than one pRNFL measure could not be fitted into the same model due to the conditional independence assumption, two LCA models were built. Four variables were included in each LCA; average and minimum macular GCIPL thicknesses by Cirrus OCT and BCVA were present in both models; temporal pRNFL thicknesses by Cirrus OCT or by Spectralis OCT were present in one model each. LCA requires tests with binary outcomes to create the model. For simplification of the analysis, white, blue, purple, and green sectors have been labeled as “normal,” and yellow and red ones as “abnormal.” For BCVA, values better than or equal to 0.3 LogMAR were labeled as normal and those worse than 0.3 as “abnormal.” BCVA was included in the model in order to provide a functional outcome that could help better define the latent class “retinal thickness change.” Temporal pRNFL was selected as it was the quadrant with a higher frequency of pRNFL thinning and abnormal results in previous studies [4, 20–23].

LCA was performed using TAGS software implemented in R version 2.2 (R Development Core Team and R Foundation for Statistical Computing, 2005). The fit of LCA model for the assumption of conditional independence was performed through the goodness-of-fit test followed by the evaluation of residual correlations between tests.

## 3. Results

Seventy RRMS patients and seventy age- and gender-matched healthy controls were enrolled in the study. All participants were of Caucasian descent.  Table 1 summarizes the demographic and clinical characteristics of the participants.

Overall, average pRNFL and temporal quadrant pRNFL thickness by both Cirrus and Spectralis OCTs were significantly lower in both ON and non-ON RRMS eyes compared to healthy eyes (*P* < 0.001). Similarly, average, minimum, and each of the 6 sectors GCIPL thicknesses yielded by Cirrus were significantly lower in RRMS compared to healthy eyes in both ON and non-ON eyes (*P* < 0.001).

All these measurements were significantly lower in eyes with a prior history of ON compared to non-ON eyes (*P* < 0.001).

Table 2 shows the percentage of abnormal color-coded measurements (defined as red or yellow color codes) obtained by GCIPL and pRNFL analysis in healthy and RRMS patients. Abnormal results were significantly more common in ON and non-ON RRMS eyes versus healthy eyes and in eyes with ON antecedent versus those without this antecedent in RRMS patients.

**Figure 2:**
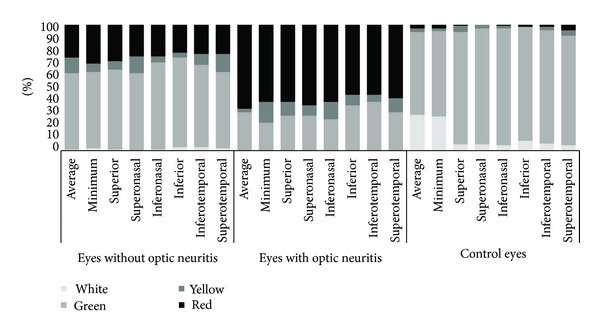
Comparison of the color scale frequency for each sector using ganglion cell-inner plexiform layer analysis with Cirrus optical coherence tomography among eyes with and without optic neuritis in relapsing-remitting multiple sclerosis patients and from healthy control subjects. Black represents eyes classified as red (below percentile 1); dark gray represents eyes labeled as yellow (below percentile 5); light gray represents green (between percentiles 5 and 95), and white represents white (above percentile 95).

Overall, the highest abnormal percentage was observed in minimum (47.8%) followed by average (46.4%) GCIPL analysis. The sector in GCIPL test showing the highest abnormality rate was the superonasal (47.1%) followed by superotemporal sector (45.7%). The abnormality rates were significantly higher in eyes with a prior ON compared to non-ON eyes (Figure 2).

Using Cirrus OCT, average GCIPL was altered more frequently than average pRNFL in eyes of patients with RRMS (46% versus 33%, resp.; *P* < 0.001).

In a subgroup analysis comparing abnormal results between GCIPL and pRNFL by ON antecedent, average and minimum GCIPL measurements yielded the highest abnormal results for both ON and non-ON eyes (Table 2).

Table 3 shows the estimated sensitivity and specificity to detect retinal thickness changes by OCT with the two LCA models. The test for evaluating the fit of the model with conditional independence (goodness-of-fit test) proved to be adjusted (*P* value = 0.938 for model A and 0.836 for model B). The residual correlations between tests were randomly distributed around 0.

Both GCIPL measurements appeared to be more sensitive and specific than temporal pRNFL thickness measured by Cirrus or Spectralis OCT (Table 3). Estimated sensitivities using Cirrus were 96.34%, 98.43%, and 58.41% for average, minimum GCIPL, and temporal pRNFL, respectively. Using Spectralis, estimated sensitivity for temporal pRNFL was lower (69.69%) than for Cirrus GCIPL measurements (average: 97.15% and minimum: 96.41%).

Importantly, average GCIPL thickness was significantly more sensitive than temporal pRNFL by Cirrus (*P* < 0.05), and minimum GCIPL was significantly more sensitive than temporal pRNFL by Spectralis for the detection of retinal thickness changes in RRMS (*P* < 0.05). The model appeared to be robust, as sensitivities and specificities for both GCIPL measurements and BCVA were similar in both models.

Abnormal results in average GCIPL thickness in RRMS patients were independently associated to age in years (OR = 0.942, *P* = 0.030), years since the diagnosis of RRMS (OR = 1.185, *P* = 0.002), and ON antecedent (OR = 4.123, *P* = 0.001), after correcting by sex and intereye correlation using binary logistic generalized estimating equations (*N* = 140).

Generalised estimating equations accounting for sex, age, and within-patient intereye correlation were used to measure standardised correlations between pRNFL and GCIPL thicknesses and disease duration, EDSS, and visual function parameters (Table 4).

Average pRNFL thickness measured with Cirrus (*β* = −0.233; *P* = 0.031) and Spectralis (*β* = −0.228; *P* = 0.025) OCTs, temporal (*β* = −0.261; *P* = 0.017) and papillomacular bundle (*β* = −0.275; *P* = 0.006) pRNFL thickness measured with Spectralis OCT, average GCIPL thickness (*β* = −0.262; *P* = 0.026), and minimum GCIPL thickness (*β* = −0.299; *P* = 0.008) correlated inversely with disease duration.

A strong, positive correlation was observed between average GCIPL thickness and pRNFL thickness using Cirrus (*β* = 0.692; *P* < 0.001) and Spectralis (*β* = 0.642; *P* < 0.001) OCTs.

After adjusting by age, sex, and within-patient intereye correlation, Cirrus GCIPL average and minimum measures showed a weak but significant correlation with EDSS only in eyes with ON antecedent (Table 4).

## 4. Discussion

Loss of RNFL is a well-documented structural marker of axonal degeneration in the eyes of patients with multiple sclerosis with and without a history of ON [8, 24–26]. Historically, OCT studies in multiple sclerosis have focused mostly on the RNFL, but retinal ganglion cell neuronal loss may also be implicated in the pathogenesis of visual dysfunction in MS [10].

To the best of our knowledge, apart from direct comparisons between pRNFL and GCIPL measurements in RRMS and healthy eyes [7–10], there is no information about the sensitivity and specificity of the different measurements to detect retinal thickness changes in RRMS patients.

Although strong positive correlation was found between GCIPL and pRNFL thickness values, average and minimum GCIPL measures showed higher sensitivity and specificity than temporal pRNFL measures. Remarkably, average GCIPL showed a significantly better sensitivity than temporal pRNFL by Cirrus, and minimum GCIPL showed a significantly better sensitivity than temporal pRNFL by Spectralis for the detection of retinal thickness changes in RRMS.

In agreement with previous studies [21], we found that eyes from patients with RRMS had significant thinning in average and temporal quadrant pRNFL values by Cirrus and Spectralis OCT, and, in each of six sectors, average and minimum GCIPL results obtained by Cirrus, when compared to healthy eyes (Table 1). This was true for eyes with and without ON antecedent. Nevertheless, eyes with ON history showed greater pRNFL and GCIPL thinning than eyes without ON antecedent [10] (Table 1).

In a previous study [4] analyzing the color-code classification of pRNFL in RRMS patients, we identified the temporal quadrant to be the most abnormally color-coded by both Cirrus and Spectralis. Additionally, temporal pRNFL quadrants were abnormally color-coded more frequently in ON eyes than in non-ON eyes by both devices, Cirrus and Spectralis.

The abnormality rate in temporal pRNFL color code in eyes with previous history of ON was 66.7% by Cirrus and 61.1% by Spectralis. The current study agrees with previous reports that eyes from patients with RRMS exhibit a significant thinning of the pRNFL and GCIPL compared with the healthy eyes [7, 10, 11] and that pRNFL thinning in RRMS patients typically occurs in the temporal sector [4, 20–23, 27–29].

As expected, the sector showing the highest abnormality rate in GCIPL test was the superonasal (47.1%).

Unsurprisingly, GCIPL thinning showed an association with disease duration and ON antecedent in eyes of patients with RRMS. GCIPL minimum represents the lowest GCIPL thickness over a single meridian crossing the annulus, which is expected to be sensitive to focal damage [30]. This measurement had previously been found to have the highest correlation with visual field pattern standard deviation in patients with chronic open angle glaucoma [31]. In our study, Cirrus GCIPL minimum measures showed a significant correlation with BCVA, mean deviation, and EDSS. Importantly, only GCIPL measures showed significant correlation with EDSS in ON eyes (Table 4).

A superior structure-function correlation between GCIPL thickness and clinical measures compared to pRNFL has been reported recently, suggesting that GCIPL analysis might be a better approach than pRNFL to examine MS neurodegeneration [11].

Previous studies have shown that GCIPL thickness can be altered in patients with RRMS [10, 32–34] and that these alterations correlated with visual function [10, 11] and central nervous system findings using magnetic resonance imaging [28]. Some of these studies have suggested that GCIPL thickness can be a more reliable measure for the detection of retinal anomalies in RRMS than pRNFL thickness [10]. Interestingly, our study demonstrates that a decrease in GCIPL thickness is more sensitive to retinal involvement in RRMS than an alteration in the temporal pRNFL.

This study has a number of limitations warranting discussion. Firstly, exact sensitivity and specificity cannot be obtained without a gold standard test. The values obtained through LCA can be useful when comparing different tests; however, the sensitivities and specificities provided are estimates. Studies with bigger sample size and including information on other diagnostic tests, such as electrodiagnostic testing and magnetic resonance imaging, could help to improve this estimation. Secondly, both eyes were included in this study. However, most MS studies published to date have included both eyes because they can be individually evaluated and do not necessarily follow the same disease course [1, 4]. Additionally, generalized estimating equations were used in order to account for sex, age, and within-patient intereye correlation. With these methods, information from both eyes can be used for the study, without the risk of increasing the risk of bias due to intereye correlation or increase in sample size [35].

The retinal segmentation algorithm used by Cirrus OCT combines GCL and IPL, since the boundaries between these two layers cannot be visually discriminated on this device. Although Spectralis OCT has developed a specific software that provides automated differentiation and quantification of the individual retinal layers, it was not available when the data was collected. Additionally, this software does not provide comparison to a normative database, so binary outcomes necessary for LCA would not be available.

Finally, we have included only patients with relapsing-remitting multiple sclerosis; therefore, our results cannot be extrapolated to other types of multiple sclerosis or to patients with more advanced disease (mean EDSS was 2.42).

## 5. Conclusions

In conclusion, OCT GCIPL analysis is more sensitive than temporal pRNFL analysis to detect retinal thickness changes in RRMS eyes. GCIPL measures correlate better than pRNFL measures with visual function parameters such as BCVA, visual field mean deviation, or EDSS. As such, GCIPL thickness measured by retinal segmentation of OCT scans may be an ideal marker for monitoring neurodegeneration in RRMS patients.

## Figures and Tables

**Table 1:** Clinical and demographic characteristics of healthy subjects and relapsing-remitting multiple sclerosis (RRMS) patients regarding optic neuritis (ON) antecedent. Differences were tested using generalized estimating equations accounting for sex, age, and within-patient intereye correlation.

	Healthy subjects	RRMS	ON eyes	Non-ON eyes	*P* (healthy versus RRMS)	*P* (healthy versus ON eyes)	*P* (healthy versus non-ON eyes)	*P* (ON versus non-ON eyes)
	*N* = 70	*N* = 70	*N* = 36	*N* = 104				
	Mean ± SD				
Age (years)	37 ± 10	40 ± 10	42 ± 9	40 ± 11	0.077	0.008	0.077	0.302
Female (%)	40 (57%)	44 (63%)	14 (78%)	30 (58%)	0.387	0.627	0.363	0.552
LogMAR BCVA	0.00 ± 0.02	0.04 ± 0.18	0.10 ± 0.26	0.02 ± 0.14	0.006	0.023	0.115	0.083
EDSS score	N/A	2.42 ± 1.72	2.43 ± 1.61	2.41 ± 1.76	N/A	N/A	N/A	0.873
Years since diagnosis	N/A	6.82 ± 6.95	7.31 ± 6.25	6.66 ± 7.19	N/A	N/A	N/A	0.754
MD (in dB)	N/A	−2.7 ± 3.8	−3.8 ± 5.2	−2.4 ± 3.1	N/A	N/A	N/A	<0.001
Average pRNFL (Sp; in *μ*m)	99.3 ± 8.7	87.8 ± 13.8	79.6 ± 13.6	90.7 ± 12.7	<0.001	<0.001	<0.001	<0.001
Temporal pRNFL (Sp; in *μ*m)	71.4 ± 10.6	58.8 ± 16.0	50.2 ± 16.2	61.9 ± 14.8	<0.001	<0.001	<0.001	<0.001
PMB pRNFL (Sp; in *μ*m)	54.9 ± 7.6	44.4 ± 11.4	38.8 ± 11.8	46.4 ± 10.7	<0.001	<0.001	<0.001	<0.001
Average pRNFL (Ci; in *μ*m)	93.9 ± 8.5	84.9 ± 12.5	78.9 ± 13.0	87.0 ± 11.7	<0.001	<0.001	<0.001	<0.001
Temporal pRNFL (Ci; in *μ*m)	64.7 ± 9.5	54.2 ± 13.8	47.4 ± 14.0	56.6 ± 13.0	<0.001	<0.001	<0.001	<0.001
Average GCIPL (Ci; in *μ*m)	83.8 ± 5.9	72.2 ± 11.3	66.4 ± 10.9	74.3 ± 10.7	<0.001	<0.001	<0.001	<0.001
Minimum GCIPL (Ci; in *μ*m)	81.9 ± 6.1	68.1 ± 12.9	61.4 ± 13.2	70.4 ± 12.0	<0.001	<0.001	<0.001	<0.001
Superior GCIPL (Ci; in *μ*m)	80.5 ± 6.2	73.0 ± 11.7	66.7 ± 11.4	75.2 ± 11.1	<0.001	<0.001	<0.001	<0.001
Superonasal GCIPL (Ci; in *μ*m)	85.5 ± 6.3	73.0 ± 11.7	65.8 ± 10.8	75.5 ± 11.0	<0.001	<0.001	<0.001	<0.001
Inferonasal GCIPL (Ci; in *μ*m)	84.0 ± 6.3	72.0 ± 12.2	64.5 ± 11.8	74.6 ± 11.2	<0.001	<0.001	<0.001	<0.001
Inferior GCIPL (Ci; in *μ*m)	82.7 ± 6.2	71.6 ± 12.0	65.6 ± 12.5	73.7 ± 11.1	<0.001	<0.001	<0.001	<0.001
Inferotemporal GCIPL (Ci; in *μ*m)	83.8 ± 6.1	73.3 ± 11.0	68.6 ± 12.3	74.9 ± 10.1	<0.001	<0.001	<0.001	<0.001
Superotemporal GCIPL (Ci; in *μ*m)	82.4 ± 6.5	72.3 ± 10.9	67.0 ± 11.0	74.2 ± 10.4	<0.001	<0.001	<0.001	<0.001

SD: standard deviation; BCVA: best corrected visual acuity; EDSS: Expanded Disability Status Scale; MD: 24-2 SITA Standard Visual Field Mean Deviation; pRNFL: peripapillary retinal nerve fiber layer; Sp: Spectralis OCT; PMB: papillomacular bundle; Ci: Cirrus OCT; GCIPL: ganglion cell and inner plexiform layers.

**Table 2:** Number of eyes (and percentage) with abnormal results (yellow or red color-coded) in healthy and relapsing-remitting multiple sclerosis (RRMS) patients and according to optic neuritis (ON) antecedent. Statistical significance of associations was tested using generalized estimating equations accounting for sex, age, and within-patient intereye correlation.

Abnormal result	Healthy	RRMS	ON eyes	Non-ON eyes	*P* (healthy versus RRMS)	*P* (healthy versus ON eyes)	*P* (healthy versus non-ON eyes)	*P* (ON versus non-ON eyes)
	*N* = 140	*N* = 140	*N* = 36	*N* = 104				
Average pRNFL (Sp)	5 (3.6)	49 (35.0)	20 (55.6)	29 (27.9)	<0.001	<0.001	<0.001	<0.001
Temporal pRNFL (Sp)	5 (3.6)	65 (46.4)	22 (61.1)	43 (41.3)	<0.001	<0.001	<0.001	<0.001
PMB pRNFL (Sp)	4 (2.9)	43 (30.7)	17 (47.22)	26 (25.0)	<0.001	<0.001	<0.001	0.012
Average pRNFL (Ci)	9 (6.4)	46 (32.9)	19 (52.8)	27 (26.0)	<0.001	<0.001	0.001	0.001
Temporal pRNFL (Ci)	2 (1.4)	49 (35.0)	24 (66.7)	25 (24.0)	<0.001	<0.001	<0.001	<0.001
Average GCIPL (Ci)	8 (5.7)	65 (46.4)	25 (69.4)	40 (38.5)	<0.001	<0.001	<0.001	<0.001
Minimum GCIPL (Ci)	7 (5.0)	67 (47.9)	28 (77.8)	39 (37.5)	<0.001	<0.001	<0.001	<0.001
Superior GCIPL (Ci)	8 (5.7)	63 (45.0)	26 (72.2)	37 (35.6)	<0.001	<0.001	<0.001	<0.001
Superonasal GCIPL (Ci)	4 (2.9)	66 (47.1)	26 (72.2)	40 (38.5)	<0.001	<0.001	<0.001	<0.001
Inferonasal GCIPL (Ci)	4 (2.9)	58 (41.4)	27 (75.0)	31 (29.8)	<0.001	<0.001	<0.001	<0.001
Inferior GCIPL (Ci)	2 (1.4)	50 (35.7)	23 (63.9)	27 (26.0)	<0.001	<0.001	<0.001	<0.001
Inferotemporal GCIPL (Ci)	6 (4.3)	55 (39.3)	22 (61.1)	33 (31.7)	<0.001	<0.001	<0.001	0.002
Superotemporal GCIPL (Ci)	12 (8.9)	64 (45.7)	25 (69.4)	39 (37.5)	<0.001	<0.001	<0.001	<0.001

pRNFL: peripapillary retinal nerve fiber layer; Sp: Spectralis OCT; PMB: papillomacular bundle; Ci: Cirrus OCT; GCIPL: ganglion cell and inner plexiform layers.

**Table 3:** Estimated sensitivity and specificity of different optic coherence tomography measures for retinal thickness changes detection in eyes of patients with relapsing-remitting multiple sclerosis (*N* = 140) and from healthy control subjects (*N* = 140) using latent class analysis. Model A: temporal pRNFL thickness as measured by Cirrus OCT; estimated prevalence of retinal thickness changes in the sample was 23.42%; 95% confidence interval (95CI) 18.50 to 29.17%. Model B: temporal pRNFL thickness as measured by Spectralis OCT; estimated prevalence of retinal thickness changes in the sample was 24.25%; 95% confidence interval (95CI) 19.26 to 30.04%.

	Sensitivity		Specificity	
	Estimate	95CI	Estimate	95CI
Model A				
Cirrus average GCIPL	96.34%	76.11 to 99.54%	97.05%	92.77 to 98.83%
Cirrus minimum GCIPL	98.43%	64.61 to 99.95%	95.81%	91.00 to 98.10%
Cirrus temporal pRNFL	58.41%	45.82 to 70.00%	93.91%	89.56 to 96.52%
Best corrected visual acuity	6.14%	2.32 to 15.30%	98.59%	95.72 to 99.54%
Model B				
Cirrus average GCIPL	97.15%	76.94 to 99.71%	97.61%	93.16 to 99.19%
Cirrus minimum GCIPL	96.41%	81.48 to 99.39%	95.43%	90.80 to 97.78%
Spectralis temporal pRNFL	69.69%	57.14 to 79.86%	92.70%	88.01 to 95.65%
Best corrected visual acuity	6.08%	2.30 to 15.15%	98.55%	95.58 to 99.53%

GCIPL: ganglion cell-inner plexiform layers; pRNFL: peripapillary retinal nerve fiber layer.

**Table 4:** Standardized correlation coefficients between OCT measurements and neurologic and visual function parameters in eyes of patients with relapsing-remitting multiple sclerosis, calculated using generalized estimating equations accounting for sex, age, and within-patient intereye correlation (*n* = 140).

	BCVA	MD	EDSS	Disease duration
Cirrus average pRNFL	0.286∗	0.418∗	−0.014	−0.233^‡^
Cirrus temporal pRNFL	−0.013	0.062	0.001	−0.141
Spectralis average pRNFL	0.314∗	0.360∗	0.033	−0.228^‡^
Spectralis temporal pRNFL	0.122^‡^	0.268^†^	−0.101	−0.261^‡^
Spectralis PMB pRNFL	0.125^‡^	0.209^‡^	−0.066	−0.275^†^
GCIPL average	0.226∗	0.513∗	−0.178	−0.262^‡^
GCIPL minimum	0.204^†^	0.412∗	−0.161	−0.299^†^

Non-ON eyes (*N* = 104)				
Cirrus average pRNFL	−0.019	0.098	0.033	−0.253^‡^
Cirrus temporal pRNFL	−0.270^‡^	−0.030	−0.046	−0.146
Spectralis average pRNFL	0.210∗	0.145	0.109	−0.274^‡^
Spectralis temporal pRNFL	−0.025	0.093	−0.092	−0.284^†^
Spectralis PMB pRNFL	0.002	0.083	−0.040	−0.332^†^
GCIPL average	−0.062	0.109	−0.113	−0.297^‡^
GCIPL minimum	0.029	0.138	−0.092	−0.314^†^

ON eyes (*N* = 36)				
Cirrus average pRNFL	0.188	0.161	−0.173	0.012
Cirrus temporal pRNFL	−0.043	0.001	0.105	−0.013
Spectralis average pRNFL	0.175	0.149	−0.151	0.109
Spectralis temporal pRNFL	0.112	0.030	−0.195	0.065
Spectralis PMB pRNFL	0.059	−0.022	−0.120	0.097
GCIPL average	0.145	0.133	−0.429^†^	−0.079
GCIPL minimum	0.144	0.120	−0.421^‡^	−0.227

*P < 0.001; ^†^ *P* < 0.01; ^‡^ *P* < 0.05.

OCT: optic coherence tomography; BCVA: best corrected visual acuity; MD: Goldman 24-2 SITA standard visual field mean deviation; EDSS: Expanded Disability Status Scale; pRNFL: peripapillary retinal nerve fiber layer; PMB: papillomacular bundle; GCIPL: ganglion cell and inner plexiform layers; ON: optic neuritis.
